# Rhizobial diversity is associated with inoculation history at a two-continent scale

**DOI:** 10.1093/femsec/fiac044

**Published:** 2022-04-13

**Authors:** Myint Zaw, Judith R Rathjen, Yi Zhou, Maarten H Ryder, Matthew D Denton

**Affiliations:** School of Agriculture, Food and Wine, The University of Adelaide, Waite Campus, Urrbrae, SA5064, Australia; Yezin Agricultural University, Yezin, Naypyidaw 15013, Myanmar; School of Agriculture, Food and Wine, The University of Adelaide, Waite Campus, Urrbrae, SA5064, Australia; School of Agriculture, Food and Wine, The University of Adelaide, Waite Campus, Urrbrae, SA5064, Australia; School of Agriculture, Food and Wine, The University of Adelaide, Waite Campus, Urrbrae, SA5064, Australia; School of Agriculture, Food and Wine, The University of Adelaide, Waite Campus, Urrbrae, SA5064, Australia

**Keywords:** central dry zone, *nodC*, inoculation, *Mesorhizobium*, Myanmar, *nifH*

## Abstract

A total of 120 *Mesorhizobium* strains collected from the central dry zone of Myanmar were analyzed in a pot experiment to evaluate nodulation and symbiotic effectiveness (SE%) in chickpea plants. Phylogenetic analyses revealed all strains belonged to the genus *Mesorhizobium* according to 16–23S rDNA IGS and the majority of chickpea nodulating rhizobia in Myanmar soils were most closely related to *M. gobiense, M. muleiense, M. silamurunense, M. tamadayense* and *M. temperatum*. Around two-thirds of the Myanmar strains (68%) were most closely related to Indian strain IC-2058 (CA-181), which is also most closely related to *M. gobiense*. There were no strains that were closely related to the cognate rhizobial species to nodulate chickpea: *M. ciceri* and *M. mediterraneum*. Strains with diverse 16S–23S rDNA IGS shared similar *nodC* and *nifH* gene sequences with chickpea symbionts. Detailed sequence analysis of *nodC* and *nifH* found that the strains in Myanmar were somewhat divergent from the group including *M. ciceri* and were more closely related to *M. muleiense* and IC-2058. A cross-continent analysis between strains isolated in Australia compared with Myanmar found that there was little overlap in species, where Australian soils were dominated with *M. ciceri*, *M. temperatum* and *M. huakuii*. The only co-occurring species found in both Myanmar and Australia were *M. tamadayense* and *M. silumurunense*. Continued inoculation with CC1192 may have reduced diversity of chickpea strains in Australian soils. Isolated strains in Australian and Myanmar had similar adaptive traits, which in some cases were also phylogenetically related. The genetic discrepancy between chickpea nodulating strains in Australia and Myanmar is not only due to inoculation history but to adaptation to soil conditions and crop management over a long period, and there has been virtually no loss of symbiotic efficiency over this time in strains isolated from soils in Myanmar.

## Teaser

Mesorhizobia isolated from Myanmar soils, where there has been little inoculation, show more genetic diversity compared to Australia where there is frequent inoculation of chickpea crops.

## Introduction

In Myanmar, pulses are currently grown on over 4 × 10^6^  hectares (21% of agricultural lands) with an annual production of 4,648,000 MT (MOALI 2020). Myanmar accounts for 5% of the world pulse cultivated area and 6% of their production (Soe and Kyaw 2016). Chickpea is mainly grown in the central dry zone (CDZ) of Myanmar by small-holder farmers and is sown on residual moisture as a second crop grown after rice in the postmonsoon season from October to November and harvested in February–March (Herridge et al. 2019). Chickpea has been cultivated in Myanmar for an unknown period of time and was likely introduced historically from India, where chickpea cultivation has been traced back to the Bronze Age (Redden and Berger 2007).

Although chickpea is an important crop in Myanmar, there are very few, if any, inputs into the growth of this crop, including inoculation. Currently, there is no established supply chain for rhizobial inoculants in Myanmar (Denton et al. 2017), although some attempts have been made to produce inoculants on a very small scale using resident strains (Than 2010). Despite the lack of inoculation, chickpea plants in Myanmar can be well-nodulated.

Chickpea is a highly specific host that can be nodulated by members of a single genus, *Mesorhizobium*, strains of which contain highly specific symbiosis genes (Laranjo et al. 2008). These strains were probably not present in Myanmar before the introduction of chickpeas. The original species of chickpea-nodulating rhizobia (*M. ciceri* or *M. mediterraneum*) that evolved in southeastern Turkey have spread throughout the world (Nour et al. 1995, Greenlon et al. 2019) and may have come to Myanmar with seeds/soil mixture during chickpea introduction from India (Rai et al. 2012). A study has investigated the diversity of a limited number of strains isolated from chickpea nodules in Myanmar and found three main *Mesorhizobium* species: *M. muleiense*, *M. tianshanense* and *M. plurifarium* (Soe et al. 2020). It is possible that Myanmar soils already had high populations of resident rhizobial strains that have acquired symbiotic ability through horizontal gene transfer (HGT) with exotic chickpea symbionts (Bouznif et al. 2019). These resident rhizobia have likely evolved with chickpea plants over a long period of cultivation and may have adapted to the Myanmar environments.

*Mesorhizobium* species contain strain-specific symbiosis islands that have different structures depending on the geography and host plants (Sullivan et al. 2002, Uchiumi et al. 2004, Kasai-Maita et al. 2013, Greenlon et al. 2019, Perry et al. 2020). The symbiosis islands contain conserved regions with symbiotic genes (*nod* or *nif*; Greenlon et al. 2019, Perry et al. 2020). In particular, the *nodC* gene is essential for nodulation in all rhizobia (Laranjo et al. 2008). In symbiotic genes, nucleotide insertion events may impact on bacterial evolution including mutations that are deleterious, neutral, or beneficial for effective symbiotic ability of rhizobia (Siguier et al. 2014, Vandecraen et al. 2017, Perry et al. 2020, Consuegra et al. 2021, Arashida et al. 2022). Given that there have been few inoculation events (or new introductions of symbiosis genes in native rhizobial populations), changes in conserved regions of *nod* genes over time may have occurred in Myanmar.

The diversity of *Mesorhizobium* has been reported within countries, but there has been very little comparison of strains between countries. Previous studies found that *M. ciceri* and *M. mediterraneum* are ubiquitous in most countries including Spain (Nour et al. 1994a, 1995), Portugal (Laranjo et al. 2004), Morocco (Maatallah et al. 2002), Tunisia (L'taief et al. 2007), India (Zafar et al. 2017) and Australia (Elias and Herridge 2015). In China, *M. ciceri* and *M. mediterraneum* were not identified (Zhang et al. 2012a,b, 2017, 2018a). In countries where chickpea has been introduced relatively recently and grown with frequent rhizobial inoculation (USA, Canada, and Australia), the majority of rhizobial strains isolated from the field are closely related to, but distinct from, the inoculant strains (e.g. *M. ciceri* strain CC1192 in Australia; Greenlon et al. 2019, Zaw et al. 2021). In contrast, a broader range of *Mesorhizobium* species was found in countries with a long history of chickpea cultivation such as India, Ethiopia and Morocco where rhizobial inoculation is rare or absent. Resident *Mesorhizobium* strains in Myanmar have experienced unique selective pressure such as cropping, and inoculation history compared with other countries and are likely to have contrasting genetic and symbiotic diversity compared with other countries where rhizobial diversity and ecology have been extensively studied.

The objectives of this study were, therefore, (1) to analyze the phylogenetic diversity of rhizobia in Myanmar chickpea-growing soils to provide a more detailed picture of the diversity of chickpea nodulating rhizobia, particularly in comparison with Australia and (2) to identify highly adapted strains with high N fixation capacity as potential inoculants in Myanmar farming systems for improving crop productivity and soil health.

## Materials and methods

### Sample collection and pH assessment

Soil samples were collected from 103 chickpea growing fields in the Mandalay, Sagaing and Magway regions of Myanmar during 2018 (Figure S1 and Table S1, Supporting Information). Soil cores were taken from a depth of 15 cm from five randomly selected points per field using a clean, sterilized spade and mixed thoroughly to make a composite sample. About 200 g of composite soil was collected from each site and placed in separate plastic bags and imported into Australia under a quarantine permit. For soil pH measurements, 5 g of dry soil was mixed with 25 ml 0.01 M CaCl_2_ and shaken on a bench-top shaker for 1 h. The soil suspension was then left to settle for 30 min to allow sedimentation before conducting pH measurement.

### Rainfall and temperature in the CDZ

Weather data for the CDZ of Myanmar (Figure S1, Supporting Information) was obtained from the Department of Meteorology and Hydrology, Naypyidaw, Myanmar. In the CDZ, chickpea is grown after monsoon rice or other lowland crops in late October or early November using residual soil moisture (Cornish et al. 2018, Herridge et al. 2019). Average monthly rainfall and temperature data for the CDZ during 2018 indicate the environmental conditions for chickpea cultivation from November to March (Figure S2, Supporting Information). The sampling sites received between 100 and 170 mm precipitation in the month of October prior to sowing chickpea and sampling of soils for this study. Rainfall distribution in the CDZ is erratic but typically highest in the May–July monsoon, with further (postmonsoon) seasonal rainfall through to October. The temperature is high (around 30 to 45°C) throughout the year (Cornish et al. 2018). There is very little rain in the winter dry season when chickpeas are grown.

### Isolation, authentication, and culture conditions of rhizobial strains

Infectivity of rhizobia was tested by growing chickpea plants in sterile pots containing 400 g of sterilized coarse sand (2–3 mm diameter) moistened with 75 ml of McKnight's N-free nutrient solution (McKnight 1949) and with a layer of collected Myanmar chickpea-growing soil (20 g), covering the sand that was then overlayed with a shallow layer of sterile sand. Chickpea cv. Hattrick seeds were sterilized in 70% ethanol for 30 s and rinsed five times with sterile water before three seeds were sown per pot. After germination, the seedlings were thinned to one plant per pot and a 2-cm layer of sterile plastic beads was added to the surface. Pots without soil were sown with chickpea seeds to check for cross-contamination. A total of 20 ml of sterile McKnight's nutrient solution was added every 3 d. The plants were kept at 20–30°C with regular sunlight in a quarantine glasshouse during winter.

Plants were removed from the pots after 5 weeks and nodules were collected and stored at −20°C. A total of three to four nodules were selected from each plant and were surface sterilized in 70% ethanol for 30 s and 6% NaOCl for 1 min and rinsed six times in sterilized distilled water. The nodules were then crushed aseptically in 100 µl of water with a sterile plastic rod. A loopful of the nodule suspension was streaked on yeast mannitol agar (YMA) plates. Single colonies of bacterial strains were selected after incubation at 28°C for 5 d and purified by subculturing through several cycles by selecting and streaking from single colonies.

For authentication as chickpea rhizobia, growth pouches (18 × 16.5 cm CYG seed germination pouches, Mega-International of Minneapolis, USA) were moistened with 20 ml of McKnight's N-free nutrient solution and sterilized by autoclaving. Chickpea seeds were surface sterilized with 70% ethanol for 20 s and three seeds were placed in each pouch. After germination, plants were thinned to one seedling per pouch and inoculated with 1 ml of inoculum (approximately 10^9^ cells ml^–1^). The seedlings were supplied with sterile McKnight's nutrient solution when necessary. A total of 3 weeks after inoculation, nodulation was assessed, rhizobia were reisolated and pure cultures stored in 25% glycerol solution at −80°C.

### Symbiotic effectiveness

The 120 test strains and *M. ciceri* CC1192, the Australian commercial chickpea inoculant strain (as a positive control) were streaked on YMA plates and incubated at 28°C for 5 d. A loopful from a single colony was transferred to yeast mannitol broth (YMB) and incubated on rotary shaker (200 r/m) at 28°C for 48 h to obtain approximately 10^9^ cells ml^–1^. A total of 1 ml of inoculum was added at the base of 5-day-old seedlings grown in sterilized sand–vermiculite media in plastic pots (120 × 75 mm). Plants were supplied with an equal amount of N-free McKnight's nutrient solution and supplemented with sterilized distilled water as necessary. Inoculation with strain CC1192 and an uninoculated treatment were included as positive and negative controls. The experiment was laid out in a randomized complete block design with three replications. Plants were harvested 50 days after sowing. Nodules were removed from the roots and counted. Leaf chlorophyll content was measured using a SPAD meter. Nodule and shoot material were oven-dried at 70°C for 48 h and dry weights per plant were recorded. Symbiotic effectiveness (SE) was calculated as described previously (Zaw et al. 2021).

### Temperature, salinity, and pH tolerance of rhizobial strains

Phenotypic assessments were performed on YMA plates divided into 20 equal squares. Each square was spot-inoculated with 10 µl of rhizobia suspension (10^9^ cells ml^–1^) previously grown in YMB. All assessments were done with three replicates in a completely randomized design. The plates were incubated at 28°C and bacterial growth was assessed 7 days after inoculation, except for the temperature tolerance experiment. Temperature tolerance was assessed by incubating cultures at 5, 10, 15, 20, 30, 35, 40 and 45°C. Salinity tolerance was assessed on YMA supplemented with 0, 0.5, 1, 1.5, 2, 2.5, 3, 3.5 and 4% NaCl (w/v), and pH tolerance on YMA adjusted to pH 4.2, 4.4, 4.6, 4.8, 5, 5.5, 6, 8, 8.5, 9, 9.5 and 10 with potassium citrate (16.2 g l^–1^) as the buffer and adjusted with 30% HCl or KOH solution. Strains were classified as tolerant, or intolerant, based on the presence or absence of growth.

### Statistical analysis

All quantitative data for symbiotic traits (nodulation, shoot dry weights, and leaf chlorophyll content) were analyzed by one-way analysis of variance (ANOVA) using a general linear model in Statistix software (Version 8) and mean values were compared at LSD (0.05%) level.

### PCR amplification and sequencing of rhizobial genes

A single colony of each strain was used as a source of DNA for PCR. Rhizobial cells from a single colony of freshly grown culture on YMA were transferred into a tube using a sterile pipette tip. Each tube had 12.5 µl MyFi^TM^ Mix added, 1 µl of each primer and 10.5 µl sterile water following manufacturer's instructions (Bioline, A Meridian Life Science Company, Sydney, Australia). PCR was carried out using a thermal cycler (Bioer Version 1.10, GeneWorks, Hindmarsh, South Australia). The primers and PCR conditions for core genes (16S–23S rDNA IGS*, dnaJ*, and *recA)* and symbiotic genes (*nodC* and *nifH)* are described in Table S2 (Supporting Information).

To verify the robustness of the 16S–23S rDNA-based phylogeny, six strains from different groups and strain CC1192 were selected for further phylogenetic analysis using two conserved housekeeping genes (*dnaJ* and *recA*). PCR amplification and sequencing of partial *dnaJ* and *recA* genes of the selected strains were undertaken as described by Alexandre et al. (2008) and Gaunt et al. (2001), respectively. The amplified PCR products were submitted to AGRF (Adelaide, South Australia) for sequence analysis using Sanger sequencing. A culture of the Indian chickpea rhizobial strain IC2058 (CA-181) obtained from ICRISAT, India was also included for sequencing in this study.

### Phylogenetic data analysis

Sequences were checked and edited using Molecular Evolutionary Genetics Analysis version 7 (MEGA7; Kumar et al. 2016). The sequences were compared with NCBI GenBank databases using nucleotide BLAST for preliminary identification. The sequences of reference and type strains available in the GenBank database were also included for comparison with test strains. To investigate the phylogeographic diversity, the 16S–23S rDNA IGS sequences of 77 Australian strains from a previous study (Zaw et al. 2021) and an Indian strain (IC-2058-CA181) were added to the phylogenetic analysis as compared with those of 114 Myanmar strains in this study. Multisequence alignments for all test and reference strains were generated with ClustalW in MEGA7. In this program, poorly aligned positions and divergent regions of nucleotide sequences were eliminated. The phylogenetic trees of 16S–23S rDNA IGS, *nodC* and *nifH* were constructed using the maximum likelihood method and positions with gaps in any sequence were deleted. The genetic distances were estimated using Kimura's two-parameter model (Kimura 1980). The robustness of the tree topology was computed by bootstrap analysis with 1000 replications for maximum likelihood analysis. Pairwise average nucleotide identity (ANI) of test and reference strains was calculated using the OrthoANIu tool (Yoon et al. 2017). The type strains or undefined species of *Mesorhizobium* available in the GenBank database were selected using a threshold of > 95% ANI for species delineation (Goris et al. 2007). Only reference and type strains which shared > 95% ANI with our test strains were included in the phylogenetic analyses. Approximately 800 bp sequence for *dnaJ* and 500 bp sequence for *recA* were obtained and used for multiple sequence alignment in MEGA 7 software. The sequences of *dnaJ* and r*ecA* were concatenated in MEGA7 and the phylogenetic tree was constructed using the best fitting model (Tamura-Nei model with discrete Gamma distribution) of nucleotide substitution to improve the bootstrap values (Tamura and Nei 1993, Laranjo et al. 2012). To evaluate amino acid changes in the *nodC* gene, the sequences were visually checked using chromatogram files, and aligned with the *nodC* sequence of *M. ciceri* CC1192 using Geneious® v.8.1 (Biomatters, Auckland, New Zealand).

### Nucleotide sequence accession numbers

The nucleotide sequences of 16S–23S rDNA IGS, *nodC*, and *nifH* were deposited in the NCBI GenBank database and the accession numbers are presented in Table S3 (Supporting Information).

## Results

### Sybiotic Effectiveness

**Figure 1.**
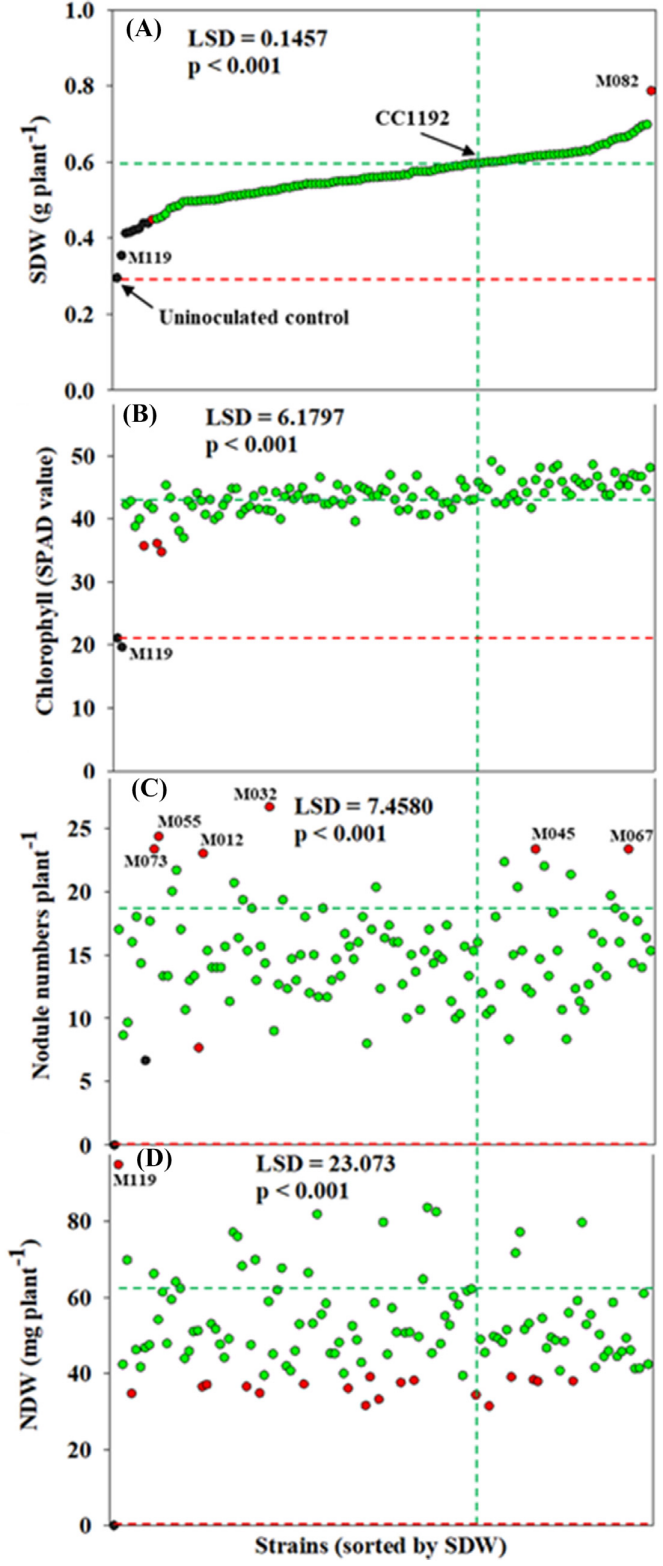
Distribution of (**A**) SDW, (**B**) leaf chlorophyll content, (**C**) nodule number per plant, and (**D**) nodule dry weight (NDW) induced by 120 test rhizobial strains and commercial strain *M. ciceri* CC1192. Strains were sorted according to their ability to enhance SDW in ascending order. Different colors of dots represent statistically different groups based on LSD (0.05) values, where black dots are strains which had a similar response to the uninoculated control, green dots represent the strains similar to the positive control (CC1192), and red dots are significantly higher or lower than both groups (black and green). The *X-* and *Y-*axis intercepts of green and red dotted lines indicate results for strain CC1192 and the uninoculated control, respectively.

Of the 120 test strains, plants inoculated with 112 strains (93%) had shoot dry weight (SDW) not statistically different to those inoculated with strain CC1192, while eight strains were not significantly different from the negative control (uninoculated). Strain M082 produced higher SDW than strain CC1192. In contrast, inoculation with strain M119 led to significantly lower SDW than most strains although it performed better than the negative control (Fig. 1A).

All test strains except M119 had greater leaf chlorophyll than the negative control. The majority of strains (97%) and CC1192 produced statistically similar chlorophyll content, while plants inoculated with strains M055, M073 and M104 had lower chlorophyll content (Fig. 1B).

Mean nodule number per plant varied from 7 (for strain M100) to 27 (for strain M032; Fig. 1C). The highest nodule dry weight (NDW) of 95 mg per plant was found in plants inoculated with strain M119, whereas the lowest NDW of 31 mg per plant was recorded from plants inoculated with M070 (Fig. 1D). Despite strain M119 forming many nodules and having a high NDW, the plants had the lowest SDW and chlorophyll accumulation efficiency (Fig. 1).

### pH, temperature, and salt tolerance of chickpea rhizobia

There was variation among the strains in ability to grow at extreme pH, temperature and salt conditions (Figure S3, Supporting Information). In general, strains were more tolerant of alkaline than acidic conditions (Figure S3a, Supporting Information). None of the strains were able to grow at > 45°C (Figure S3b, Supporting Information), but 10 strains grew at low temperature (5°C). All strains were particularly sensitive to high NaCl concentrations (Figure S3c, Supporting Information).

### Phylogeny of the 16S–23S rDNA IGS region of chickpea rhizobia

Based on the quality of sequences, 114 out of 120 strains were selected and subjected to phylogenetic analysis using the MEGA-7 program. Distantly related rhizobial genera such as *Bradyrhizobium*, *Sinorhizobium*, and *Rhizobium* were separated as an outgroup from the *Mesorhizobium* genus.

**Figure 2.**
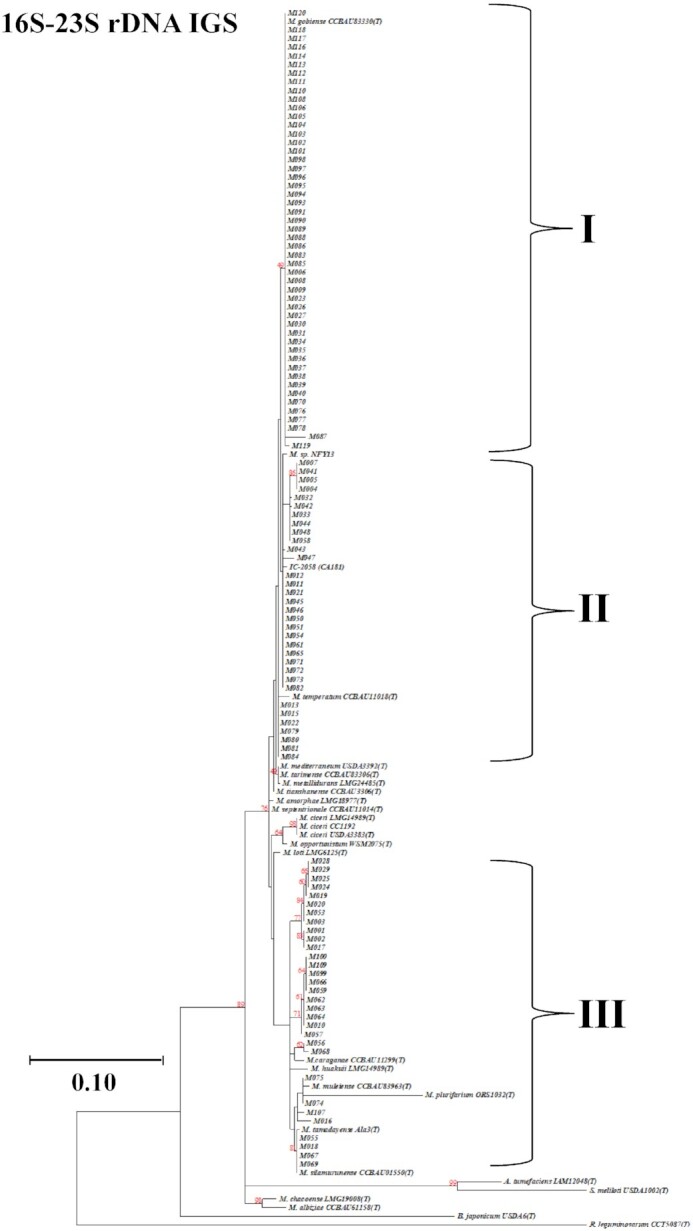
Maximum likelihood phylogenetic tree based on 16S–23S rDNA IGS sequences of 114 Myanmar rhizobial strains and reference strains. Bootstrap values were computed based on 1000 replications. The scale bar (0.10) indicates the percentage of nucleotide substitutions per site. *A*, *Agrobacterium*, *B*, *Bradyrhizobium*, *M*, *Mesorhizobium, S, Sinorhizobium*, and *R, Rhizobium*.

Approximately half of the test strains (50) were clustered into Group I that had 100% ANI with *Mesorhizobium gobiense* (Fig. 2). This group contained two acid-tolerant strains (M094 and M113) collected from the Magway region. *Mesorhizobium gobiense* was the dominant species in all sampling regions (Sagaing, Mandalay, and Magway) in this study.

A total of 33 strains belonging to Group II had 95%–100% ANI with *M. temperatum*, *M. mediterraneum*, *M. tarimense*, *M. tianshanense*, *M. metallidurans* and an Indian strain IC-2058 (CA-181). Among these, 14 strains shared identical 16S–23S rDNA IGS sequences (100% ANI) with the Indian strain, while seven strains of the broader group were more closely related to *M. temperatum*. Group II also contained strains with the highest SE (e.g. M045 and M082), acid tolerance (M065) and heat tolerance to 45°C (M021, M043, M061, and M082; Fig. 2; Figure S3, Supporting Information).

Group III contained 33 strains, which shared 95%–100% ANI values between the strains within this group. Among them, four strains (M018, M055, M067 and M069) shared 100% ANI with *M. silamurunense* and *M. tamadayense*. A total of four strains (M016, M074, M075 and M107) were most closely related to *M. plurifarium* and *M*. *muleiense*, with 95%–97% ANI. The remaining strains in Group III were more closely related to *M. huakuii* and *M. caraganae* with 95%–96% ANI. This group contained strains with varying effectiveness, and slightly more divergent 16S–23S rDNA IGS regions, as compared with Groups I and II. Strains belonging to Group III were isolated from Mandalay, Sagaing, and Magway regions and had varying SE from inferior (e.g. M055) to equal (e.g. M075) SE, relative to strain CC1192.

### Phylogeny of symbiosis-related genes

**Figure 3.**
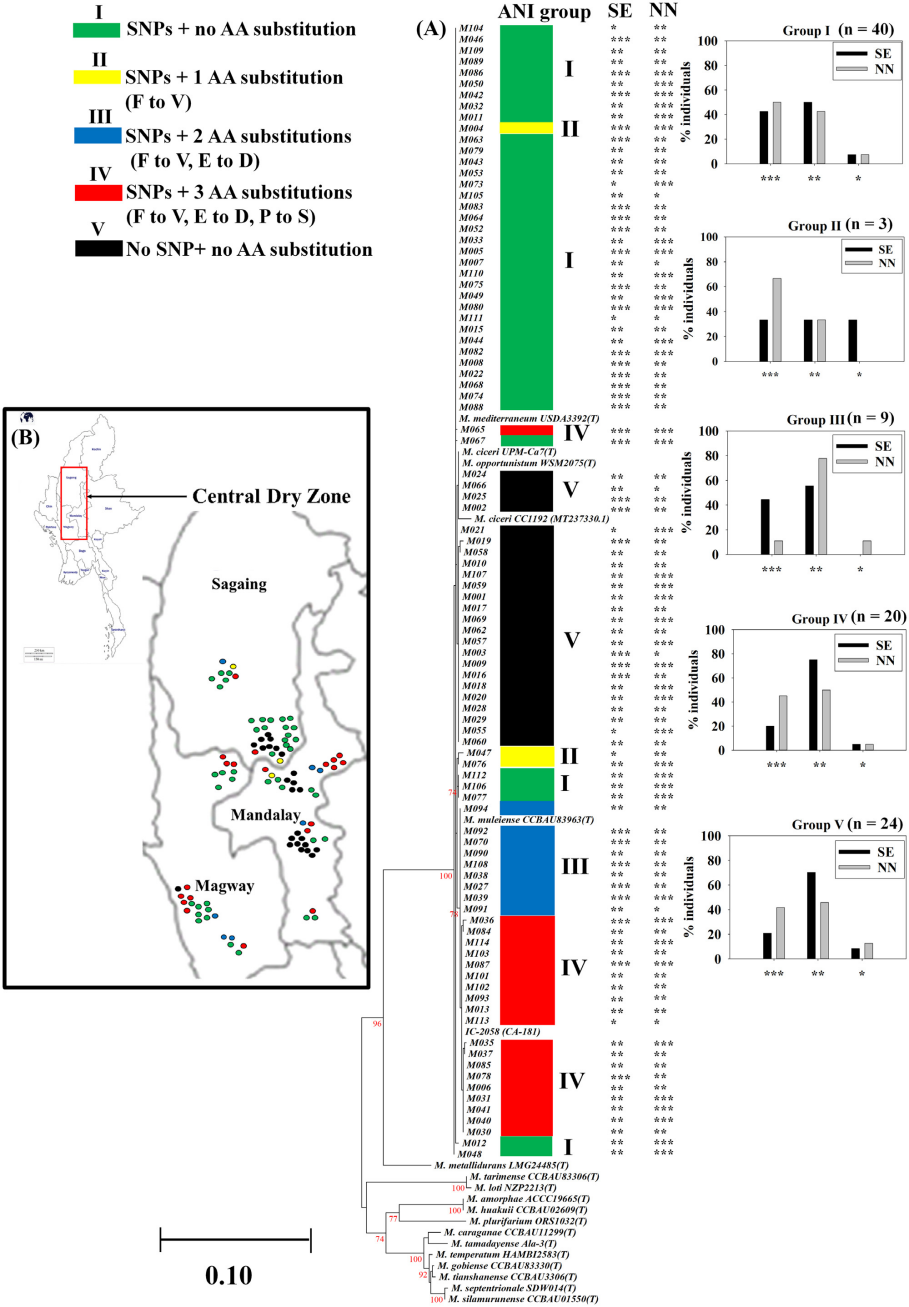
**(A)** Phylogenetic trees based on partial sequences of *nodC* with different SNPs and amino acid substitutions as compared with the chickpea symbiont, *M. ciceri* strain CC1192, which is the Australian commercial inoculant. The phylogenetic tree was generated using the Maximum Likelihood method based on the Kimura two-parameter model in MEGA7. Bootstrap values were computed based on 1000 replications. The scale bar (0.1) represents the percentage of nucleotide substitutions per site. All positions containing gaps and missing data were eliminated. **(B)** The distribution of strains from five different ANI groups in association with sample collection sites. *M*, *Mesorhizobium*, SE relative to CC1192 (***> 100%, **80%–100%, and *< 80%), NN, nodule numbers (***> 15, **10–15, and *< 10), AA, amino acid, D, Aspartic acid (Asp), E, Glutamic acid (Glu), F, Phenylalanine (Phe), P, Proline (Pro), S, Serine (Ser), V, Valine (Val), and *n*, number of strains.

**Figure 4.**
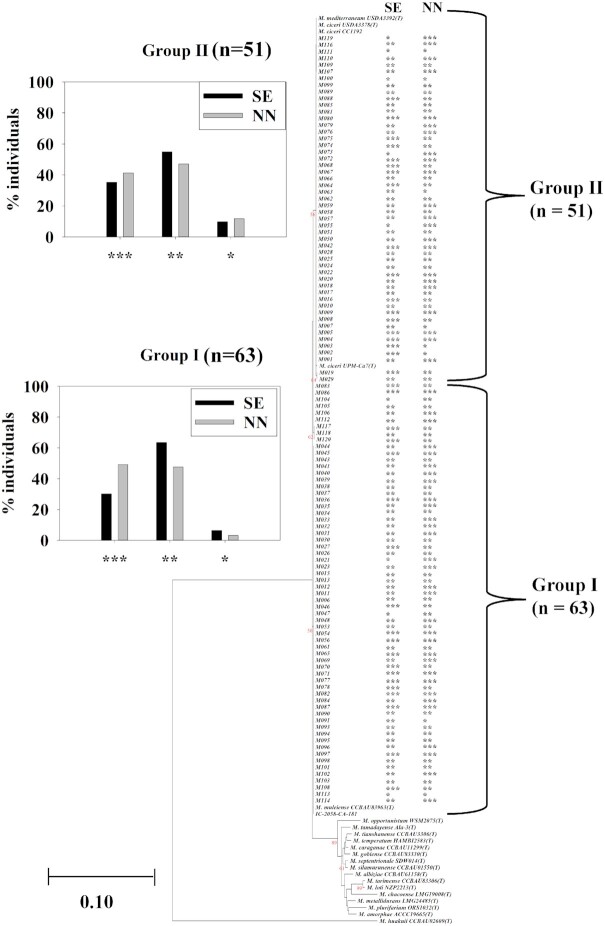
Phylogenetic tree based on partial sequences of *nifH* showing the relatedness of this gene among chickpea rhizobial strains and recognized species of *Mesorhizobium*. The phylogenetic tree was generated using the Maximum Likelihood method based on the Kimura two-parameter model in MEGA7. Bootstrap values were computed based on 1000 replications. The scale bar (0.1) represents the percentage of nucleotide substitution per site. All positions containing gaps and missing data were eliminated. *M*, *Mesorhizobium*, SE relative to CC1192 (***> 100%, **80%–100%, and *< 80%), NN, nodule numbers (***> 15, **10–15, and *< 10), *n*, number of strains.

The grouping of strains in both *nodC* and *nifH* phylogenetic trees was highly similar (Figs 3 and 4). All test strains had similar *nodC* gene sequences (nucleotide position between 490 and 1000) with 99%–100% ANI to other chickpea symbionts (Fig. 3A). Partial sequences of the gene were aligned with the translated *nodC* sequence of *M. ciceri* CC1192 (MT237330.1) and subdivided into five different groups. The groups are outlined in Table 1. Most of the strains (75%) had eight SNPs and one (Group II), two (Group III), or three (Group IV) amino acid substitutions. The strains in group IV had the greatest differences in *nodC* sequences, with three amino acid substitutions: Pro-775-Ser, Glu-981-Asp, and Phe-988-Val relative to CC1192. The *nodC* sequences of Indian strain IC-2058 (CA-181) and *M. muleiense* were closely related to those of Group IV and III, respectively. Analysis of the groupings and SE and nodule numbers showed that there was no difference among the groups according to SNPs and amino acid substitutions in nodulation or SE (Fig. 3A). Group I, which has three amino acid substitutions compared with CC1192, had the most strains with high and intermediate SE and nodule numbers, which has no amino acid substitutions with CC1192. Group IV, which has three amino acid substitutions, showed a profile of SE and NN similar to CC1192.

**Table 1.** Comparison amino acid substitution in highly conserved regions of *nodC* genes of Myanmar chickpea rhizobia relative to that of *M. ciceri* CC1192.

	**Amino acid number**							
	543	687	775	849	895	981	988	996
	**Amino acid**							
	Arg	Gln	Pro	Arg	Leu	Glu	Phe	His
**Consensus sequence**								
**ANI group**	CGA	CAG	CCA	CGG	CTG	GAG	TTT	CAT
I (40)	CGG	CAA	TCA	CGA	TTG	GAT	GTT	CAC
II (3)	CGG	CAA	TCA	CGA	TTG	GAT	GTT(Val)	CAC
III (9)	CGG	CAA	TCA	CGA	TTG	GAT(Asp)	GTT(Val)	CAC
IV (20)	CGG	CAA	TCA(Ser)	CGA	TTG	GAT(Asp)	GTT(Val)	CAC
V (24)	-	-	-	-	-	-	-	-

A red coloured letter represents a single nucleotide polymorphism compared with the consensus nucleotide sequence of CC1192, and a dash (-) indicates an identical codon to sequence of CC1192. Letters in the bracket (e.g. Val and Asp) indicates the amino acid substitution has occurred. The number in the bracket indicates the number of strains in which specific mutation(s) or amino acid substitution(s) were identified.

The *nifH* based phylogeny showed that all test strains carry highly similar nucleotide sequences for this gene and were grouped into a main single cluster with 99%–100% ANI (Fig. 4).

### Comparative sequence analysis of 16S–23S rDNA IGS, *nodC*, and *nifH* genes of chickpea rhizobial strains from both Myanmar and Australia

**Figure 5.**
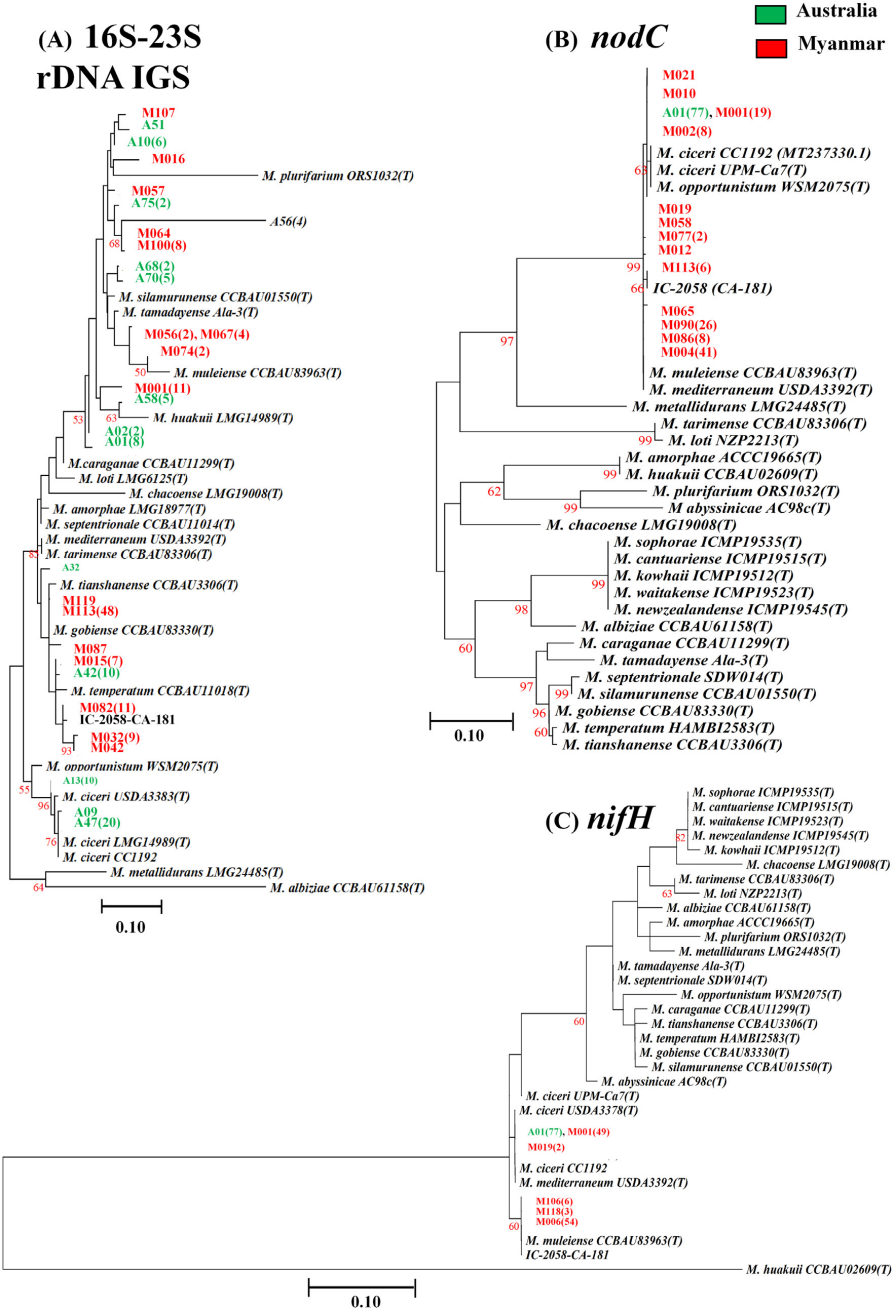
Comparative analysis of **(A)** 16S–23S rDNA IGS, **(B)***nodC*, and **(C)***nifH* sequences of 114 Myanmar strains, 77 Australian strains, and reference strains. The maximum likelihood phylogenetic tree was constructed using Kimura two-parameter model in MEGA7. Bootstrap values were computed based on 1000 replicates. The scale bar (0.10) indicates the percentage of nucleotide substitutions per site. *A*, *Agrobacterium*, *B*, *Bradyrhizobium*, *M*, *Mesorhizobium*, *S*, *Sinorhizobium*, and *R*, *Rhizobium*. Strains from Australia (green color) and Myanmar (red color) were designated as A and M, respectively (e.g. A01 and M001). The numbers in the brackets indicate the number of strains with identical nucleotide sequences (> 99% ANI).

Comparative phylogenetic analysis of 16S–23S rDNA IGS sequences of strains isolated from soils in Myanmar and Australia revealed that most Myanmar strains (68%) were closely related to the Indian strain IC-2058 (CA181), which itself has high sequence identity (99%–100% ANI) to *M. gobiense* and *M. tianshanense* (Fig. 5A). In contrast, most Australian strains (40%) shared highly similar sets of 16S–23S rDNA IGS sequences with the original chickpea symbiont *M. ciceri*, including Australian commercial inoculant strain *M. ciceri* CC1192. Other than *M. ciceri*, about 13% of Australian strains shared 100% ANI with *M. temperatum*. In general, Australian strains were related to well-known chickpea rhizobia (*M. ciceri*), while Myanmar strains were more closely related to rarely described chickpea symbionts such as *M. gobiense, M. tianshanense, M. tamadayense* and *M. silamurunense*. There was little overlap in species between two countries, where Australian soils were dominated by strains related to *M. ciceri*, *M. temperatum* and *M. huakuii*, while the only species found in both countries were *M. silamurunense* and *M. tamadayense* (Fig. 5A).

Although symbiosis gene-based phylogenies incorporating both Australian and Myanmar strains were highly similar, sequence analysis revealed that most Myanmar strains have some changes in the *nodC* and *nifH* sequences (Fig. 5B and C). Only 21 (20%) and 51 (44%) Myanmar strains had 100% ANI with *nodC*and *nifH* of *M. ciceri* strains including CC1192. Most Myanmar strains (80%) shared highly similar *nodC* sequences (99%–100% ANI) with *M. tamadayense* and *M. opportunistum*, *M. mediterraneum*, *M. muleiense* and Indian strain IC-2058-CA-181 (Fig. 5A). The *nifH* genes of more than half of the Myanmar strains (56%) were most closely related to the *nifH* of *M. muleiense*, *M. tarimense* and *M. tianshanense* and strain IC-2058-CA181 with 99%–100% ANI (Fig. 5C). In contrast, all Australian strains tested (77) were tightly clustered together with *M. ciceri* CC1192 in both *nodC* and *nifH* phylogenies (Fig. 5B and C).

### Phylogeny of concatenated *dnaJ*-*recA* genes

The concatenated phylogenetic trees *dnaJ* and *recA* revealed that the six selected rhizobial strains from Myanmar fell into three different clusters (Figure S4, Supporting Information). The grouping of strains in concatenated *dnaJ-recA* phylogenetic trees was consistent and in agreement with that of the 16S–23S rDNA-based phylogeny. For example, the acid-tolerant strain M065 and the highly symbiotically effective strain M082 represented Group II of 16S–23S rDNA IGS-based phylogeny and were also in the same cluster in the concatenated *dnaJ-recA* phylogenetic tree (Fig. 2; Figure S4, Supporting Information). A symbiotically effective strain (M009) and two acid-tolerant strains (M094 and M113) from Group I shared 100% ANI with *M. gobiense* in all phylogenies in this study. Also, a heat-tolerant strain M075 from Group III was consistently assigned together with *M. tamadayense* in both tree topologies of 16S–23S rDNA and concatenated *dnaJ*-*recA* (Fig. 2; Figure S4, Supporting Information).

### Biogeographic distribution of selected rhizobial strains from Myanmar and Australia

**Figure 6.**
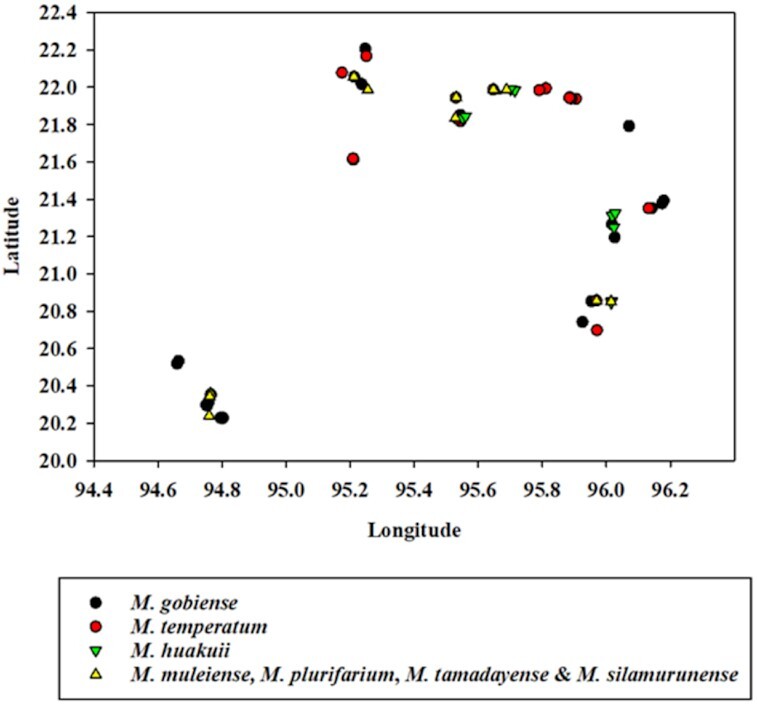
Spatial distribution of *Mesorhizobium* species in CDZ of Myanmar. Different colors and symbols in the scatter plot indicate relative distribution of mesorhizobial species to each other in space. *Mesorhizobium* species were identified based on 16S–23S rDNA phylogeny (see Fig. 5). *M, Mesorhizobium*.

A total of 79 Myanmar strains representative of different geographic locations were subjected to scatter plot analysis to investigate the spatial distribution of *Mesorhizobium* species in the CDZ of Myanmar. Figure 6 shows the spatial distribution of test strains under four main species groups representing the closely related seven identified *Mesorhizobium* species. The three main species groups of chickpea-nodulating rhizobia representing *M. gobiense*, *M. huakuii* and *M. muleiense* were distributed throughout Myanmar but *M. temperatum* was not found in the southwestern area of Magway (Fig. 6).

**Figure 7.**
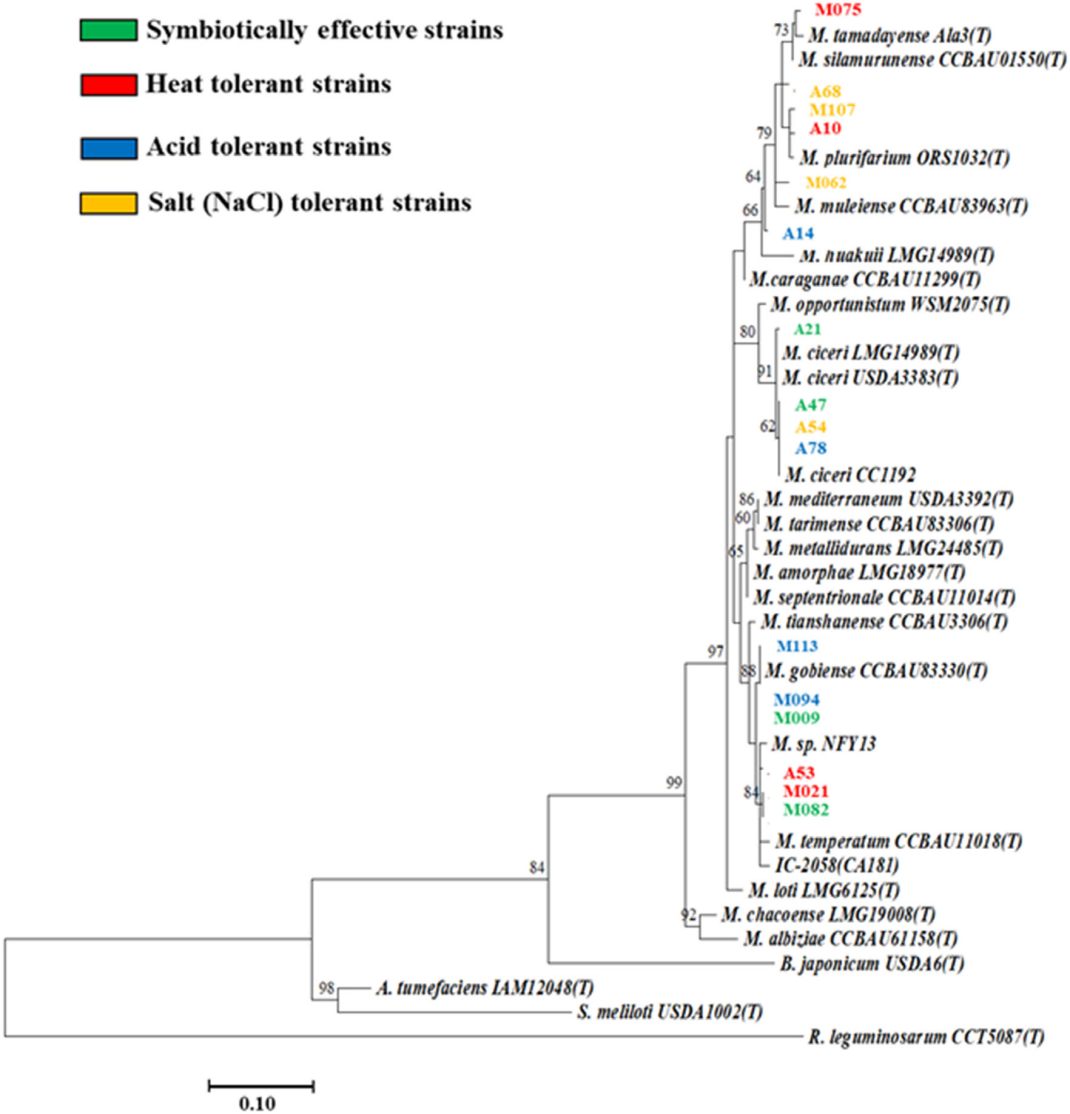
Comparative sequence analysis using16S–23S rDNA IGS of 16 selected strains with potential ecological adaptation and symbiotic traits, and CC1192. Strains from Myanmar and Australia were abbreviated as M (e.g. M113) and A (e.g. A47), respectively. Bootstrap values were computed based on 1000 replications. The scale bar (0.10) indicates the percentage of nucleotide substitutions per site. *A*, *Agrobacterium*, *B*, *Bradyrhizobium*, *M*, *Mesorhizobium*, *S*, *Sinorhizobium*, and*R*, *Rhizobium*.

A total of 16 strains from Myanmar and Australia were selected for further phylogenetic analysis based on their potential in SE, tolerance to acid, high temperature, and high salt concentration as well as their divergence in 16S–23S rDNA-based phylogeny (Figs 2 and 7). A total of two acid-tolerant strains from Myanmar (M094 and M113) were closely related to each other, while strains A68 and M062 also shared similar 16S–23S rDNA phylogeny as well as having high salt tolerance. The remaining strains did not show any strong relationship between physiological and phylogenetic relatedness.

## Discussion

### Genetic diversity of chickpea-nodulating rhizobia in Myanmar

Phylogeny of 16S–23S rDNA IGS shows that the majority of chickpea nodulating rhizobia isolated from Myanmar soils were most closely related to *M. gobiense*, *M. silamurunense, M. tamadayense* and *M. temperatum* (Fig. 2), some of which have also been found in northern China, India and Ethiopia (Table S3, Supporting Information; Zhang et al. 2012a,b, 2020, Rai et al. 2012, Muleta et al. 2022). Some of the chickpea rhizobial species such as *M. temperatum*, *M. huakuii* and *M. plurifarium* in Myanmar have also been found throughout the world (Gao et al. 2004, Alexandre et al. 2009, Elias and Herridge 2015, Tena et al. 2017). Previous studies in India found that most isolated strains were closely related to *M. ciceri* and *M. mediterraneum* (Rai et al. 2012). This is in contrast to the present study where we found the Indian strain IC-2058 (CA-181) was not closely related to those cognate species and is more closely related to the *Mesorhizobium* species found in Myanmar. No strains from Myanmar were closely related to *M. ciceri* and *M. mediterraneum*, which may be due to the lack of inoculation in the chickpea growing regions of Myanmar; it is also possible that the Myanmar strains originally came from India with the historic introduction of chickpea or are resident soil strains.

In total, three of the four main species groups of chickpea-nodulating rhizobia, *M. gobiense*, *M. huakuii* and *M. muleiense*, were distributed throughout Myanmar, but *M. temperatum* was not found in the Southwestern area of Magway (Fig. 6). In China, these four species were isolated from alkaline sandy desert soils (Han et al. 2008, Zhao et al. 2012). The soils in Myanmar range from vertisols, located mainly in the Sagaing and Mandalay regions, to sandy uplands which are commonly found in the Magway region (Herridge et al. 2019). The farming systems in Magway differ from those in the lowland central regions of the CDZ, as they are mostly rainfed and do not include rice rotations. It is possible that the *Mesorhizobium* species found in Magway require specific adaptive traits similar to those of species found in northern China, while the heavy vertisol soils differ in having less stressful conditions that are able to support a greater diversity of *Mesorhizobium* species (Greenlon et al. 2019). A study in China showed that the population composition of *M. muleiense* shifted with changing soil nutrient and pH conditions over time (Zhang et al. 2018b). Crop rotations and inputs that affect soil chemistry may also be a factor in the regional species differences found in Myanmar.

Although there has been no widespread rhizobial inoculation in Myanmar, chickpea nodulating rhizobia are abundant in cropping soils. During sample collection in Myanmar, we observed that chickpea was well-nodulated in most fields (sometimes with up to 40 nodules per plant) and the sizes of the nodules were much bigger than those observed in our pot experiments. Notably, among the 114 chickpea mesorhizobial strains obtained from geographically diverse Myanmar soils, most established effective symbioses with chickpea. Our strains were extracted from a thin layer of soil in sterilized sand, which may have led to isolation of a greater diversity of strains than those found in nodules in Myanmar, as was observed for bean and soybean in Brazil (Alberton et al. 2006), and may be relevant to this study.

### Adaptive traits of mesorhizobia in Myanmar

The comparative phylogenetic analysis of the Myanmar and Australian strains that were selected by physiological tolerance to abiotic stresses showed that the physiological attributes of strains are more likely associated with local soil environments than with phylogeny. In a previous study, *M. gobiense* and *M. tianshanense* were acid sensitive strains, and about 50% of the *Mesorhizobium*-like strains including *M. ciceri* were tolerant to acidic pH (Laranjo and Oliveira 2011), but our results suggest that the physiological attributes of rhizobial strains did not cluster according to the classification of the type strains. *M. plurifarium* is reported as acid, heat, and salt tolerant (de Lajudie et al. 1998) and was associated with salt and heat tolerance of strains from Australia and Myanmar (salt) and Myanmar (heat) but not acid tolerance in our test strains. Another two heat-tolerant reisolated strains (M021 and A53) were clustered together with *M. temperatum* (Fig. 7), which was originally isolated from *Astragalus adsurgens* in China (Table S3, Supporting Information; Gao et al. 2004) and was sensitive to high temperature (Han et al. 2008). Apart from salt and heat tolerances, which were related to genetic similarities, acid tolerance was only present in two closely related strains from Myanmar. Adaptative traits like heat and salt tolerance would explain the success of the isolated strains from their soils of origin, but there was no clear association of these traits with phylogenetic relatedness.

### Diversity of symbiotic genes in Myanmar

Although this study shows a diverse array of chickpea nodulating species present in and adapted to Myanmar soils, according to DNA sequence data of core genes, our strains contained sets of both *nodC* and *nifH* genes that were highly similar or identical to those of other chickpea symbionts found in other countries. The most likely explanations are that symbiosis genes were either dispersed to the resident mesorhizobial population through HGT (Sullivan et al. 1995, Hill et al. 2021), with the original historic introduction of chickpea into Myanmar, or became dispersed more recently through introduction on the seed from neighbouring countries, as found in recent phylogenetic study on peanut *Bradyrhizobium* species (Bouznif et al. 2019).

Studies on the *Rhizobium* and *Bradyrhizobium nod* gene have revealed that amino acid substitutions (e.g. from aspartic acid to asparagine) or deletions in the symbiosis genes can cause lower nodule numbers than nonmutant strains, and a failure to fix nitrogen (Burn et al. 1989, Göttfert et al. 1989, Krause et al. 2002, Arashida et al. 2022). In this study, we found that amino acid substitutions had no effect on either SE or nodule number per plant, even with a major amino acid substitution of phenylalanine to valine in groups II, III, and IV compared to the *nodC* protein of *M. ciceri* CC1192. The groups most divergent from the *nodC* gene sequence of CC1192 contained the Indian strain IC-2058-CA181 (Group IV) and *M. muliense* (Group III). The *nodC* sequence in groups containing the Indian strain and *M. muliense* were found to be most closely related to *nodC* genes found in rhizobia from chickpea nodules in Ethiopia (Muleta et al. 2022). A similar pattern was found with the sequences of *nifH* genes, where genetic divergence among *nifH* sequences of Myanmar strains had no effect on SE. This congruence may indicate a common origin of these symbiosis genes, or that they have acquired the same genetic differences.

A genetic diversity and gene flow study on *Mesorhizobium* strains revealed that the nodulating symbionts have evolved divergently in association with geographic distribution and have been selected by both host plant genotypes and local environments (Ji et al. 2015). In mesorhizobial strains of black locust, *Robinia pseudoacacia*, the symbiotic haplotypes were not found in different continents, but were found in multiple locations within China, indicating that there was no recent transmission of symbiotic genes (Liu et al. 2019). This finding is in agreement with the results of the present study. The heavy selection pressure by the yearly planting of chickpea crops in Myanmar would maintain symbiotic integrity of the *nodC* gene, even in the absence of HGT from a regularly applied commercial inoculant. Even though the transfer of symbiosis genes to native Myanmar strains was most likely historic and rare, there has been very little divergence relative to the symbiosis genes of inoculant species such as *M. ciceri*, and there is high nodulation of chickpea crops in Myanmar soil.

### Comparison of mesorhizobial diversity in Myanmar and Australia

Comparative sequence analysis between the chickpea nodulating strains recently reisolated from soil in Australia and in Myanmar showed that there was a much lower level of diversity among Australian reisolated strains, with the majority closely related to *M. ciceri*. This was not surprising, as Australia has a much more recent history of chickpea cultivation than Myanmar and frequent inoculation of Australian chickpea crops, leading to a greater prevalence of strains in soil that are related to the inoculant strain. The low degree of chickpea rhizobial diversity in Australia compared to a country with a very different chickpea cropping history such as Myanmar, has been observed in a comparison of strains reisolated in India, Portugal, and Ethiopia with those present in soil in the USA, Canada, and Australia (Greenlon et al. 2019).

The occurrence of strains related to species other than *M. ciceri* in Australian soils was also not comparable to the species found Myanmar. For example, a high proportion of Australian strains was closely related to *M. temperatum*, which was only found for relatively few (6%) of the Myanmar strains. The only significant overlap in species reisolated from chickpea soils between the Australia and Myanmar was found with a group related to *M. silamurunense* and *M. tamadayense*. It is likely that this lack of comparability between *Mesorhizobium* species found in Myanmar compared to Australia is not only due to inoculation history but also to crop management, inputs and resident soil species (Hirsch 1996). There may also be an effect of isolated and independent genetic changes throughout the history of chickpea cultivation in Myanmar (Liu et al. 2019), which would not be found in Australia due to the much more recent introduction of chickpea (mid-1970s).

### Symbiotic genes in Myanmar and Australia

Inoculation history is also reflected in the phylogeny of the symbiosis genes of Myanmar and Australian strains, where the *nodC* and *nifH* in reisolated Australian strains were closely related. The *nodC* sequences from most of the Myanmar strains were in a slightly different phylogenetic group (*M. muleiense* and IC-2058-CA181) from those present in the Australian strains (related to *nodC* from *M. ciceri* CC1192). A similar pattern was found with the *nifH* genes. These results support the observation that the symbiosis genes found in Myanmar soils are different than in soils where there has been frequent inoculation of commercial strains, as has occurred in Australia, where descendants of the inoculant strain were commonly found among reisolated strains (Zaw et al. 2021). A study of Ethiopian strains also uncovered this discrepancy in *nodC*/*nifH* genes, with a majority group closely clustered with *M. ciceri* and a small group also more closely related to *M. muleiense* (Muleta et al. 2022). The divergence of the sequence of the *nodC*/*nifH* genes in this study between the Australian and Myanmar strains indicates that the dispersal of symbiosis genes from a common symbiont was probably an historical event, which has not been recently repeated in Myanmar.

## Conclusion

This study has generated a detailed analysis of the diversity of chickpea-nodulating rhizobia in Myanmar. The investigation has uncovered that Myanmar is a source of genetic variation of mesorhizobia compared to Australia, where there has been frequent inoculation of the commercial strain *M. ciceri* CC1192. We have provided an insight into the ability of strains that are distantly related, to harbor similar nodulation and fixation genes and form effective symbiosis in genetically diverse backgrounds. The chickpea-nodulating rhizobia in Myanmar are more closely related to species found in India and China, which may be a function of soil adaptation or dispersal over the history of chickpea cultivation.

This study has also identified subtle changes in the symbiosis gene *nodC*, which has occurred over time without the constant introduction of these genes through inoculation. Compared with Australia, the symbiosis genes found in Myanmar have some changes in the protein sequence, which at this point have not changed SE, but further mutations may well incur penalties or even improvements in nitrogen fixation over time.

## Supplementary Material

fiac044_Supplemental_File
