# Supplementary material for: Rhizobial diversity is associated with inoculation history at a two-continent scale
Source: FEMS Microbiol Ecol. 2022 Apr 13;98(5):fiac044. doi: 10.1093/femsec/fiac044 (PMC9329089; doi:10.1093/femsec/fiac044)
Supplement: fiac044_Supplemental_File [file fiac044_supplemental_file.docx]

**Table S1**. GPS coordinates, town, region and soil pH of strain collection sites

| **Strain** | **Latitude-Longitude** | **Altitude** | **Town** | **Region** | **Soil pH**  **(CaCl_2_)** | **Strain** | **Latitude-Longitude** | **Altitude** | **Town** | **Region** | **Soil pH**  **(CaCl_2_)** |
| --- | --- | --- | --- | --- | --- | --- | --- | --- | --- | --- | --- |
| M001, M002 | 21° 50′ 7.52″ N  95° 33′ 1.55″ E | 65m | Ngazun | Mandalay | 7.04 | M062 | 21° 59′ 11.58″ N  95° 38′ 50.97″ E | 70m | Myinmu | Sagaing | 6.42 |
| M003 | 21° 50′ 37.09″ N  95° 33′ 37.99″ E | 66 m | Ngazun | Mandalay | 7.55 | M063, M064 | 21° 56′ 36.79″ N  95° 31′ 52.73″ E | 62m | Myinmu | Sagaing | 6.75 |
| M004, M005 | 21° 49′ 0.91″ N  95° 32′ 40.94″ E | 80m | Ngazun | Mandalay | 7.57 | M065 | 21° 56′ 36.48″ N  95° 31′ 48.98″ E | 62m | Myinmu | Sagaing | 7.14 |
| M006 | 21° 49′ 48.05″ N  95° 32′ 10.74″ E | 62 m | Ngazun | Mandalay | 7.63 | M066 | 21° 59′ 4.89″ N  95° 15′ 15.28″ E | 72m | Chaung U | Sagaing | 6.76 |
| M007, M008 | 21° 49′ 50.70″ N  95° 32′ 5.88″ E | 61m | Ngazun | Mandalay | 7.18 | M067, M068 | 21° 59′ 7.26″ N  95° 15′ 15.91″ E | 71m | Chaung U | Sagaing | 6.73 |
| M009 | 21° 51′ 3.22″ N  95° 32′ 39.20″ E | 60m | Ngazun | Mandalay | 7.39 | M069 | 21° 59′ 6.12″ N  95° 15′ 19.12″ E | 73m | Chaung U | Sagaing | 6.46 |
| M010 | 21° 50′ 4.33″ N  95° 31′ 43.58″ E | 59m | Ngazun | Mandalay | 6.81 | M070 | 22° 12′ 20.14″ N  95° 14′ 50.14″ E | 127m | Monywa | Sagaing | 7.75 |
| M011, M012 | 20° 41′ 53.77″ N  95° 58′ 14.92″ E | 177m | Pyawbwe | Mandalay | 7.57 | M071 | 22° 9′ 58.53″ N  95° 15′ 2.53″ E | 119m | Monywa | Sagaing | 7.61 |
| M013 | 20° 44′ 29.98″ N  95° 55′ 32.66″ E | 185m | Pyawbwe | Mandalay | 7.45 | M072, M073 | 22° 4′ 38.29″ N  95° 10′ 28.82″ E | 68m | Monywa | Sagaing | 7.72 |
| M014, M015 | 20° 51′ 12.43″ N  95° 57′ 13.83″ E | 184m | Meiktila | Mandalay | 7.67 | M074 | 22° 3′ 14.73″ N  95° 12′ 40.34″ E | 69m | Monywa | Sagaing | 7.70 |
| M016 | 20° 51′ 17.81″ N  95° 58′ 9.68″ E | 178m | Thazi | Mandalay | 6.34 | M075, M076 | 22° 3′ 16.73″ N  95° 12′ 42.69″ E | 69m | Monywa | Sagaing | 7.73 |
| M017 | 20° 51′ 8.45″ N  96° 1′ 2.92″ E | 163m | Thazi | Mandalay | 7.22 | M077, M078 | 22° 3′ 17.51″ N  95° 12′ 45.40″ E | 70m | Monywa | Sagaing | 7.75 |
| M018, M019 | 20° 50′ 59.95″ N  96° 0′ 53.77″ E | 157m | Thazi | Mandalay | 7.31 | M079 | 22° 1′ 0.88″ N  95° 14′ 9.44″ E | 67m | Chaung U | Sagaing | 7.67 |
| M020 | 20° 50′ 52.20″ N  96° 0′ 53.95″ E | 169m | Thazi | Mandalay | 6.14 | M080, M081 | 22° 0′ 56.60″ N  95° 14′ 10.08″ E | 67m | Chaung U | Sagaing | 7.51 |
| M021, M022 | 20° 51′ 22.24″ N  95° 58′ 8.75″ E | 168m | Thazi | Mandalay | 7.31 | M082 | 21° 37′ 6.72″ N  95° 12′ 32.84″ E | 62m | Yesagyo | Magway | 7.55 |
| M023 | 21° 11′ 43.15″ N  96° 1′ 34.52″ E | 123m | Wundwin | Mandalay | 7.31 | M083, M084 | 21° 37′ 5.83″ N  95° 12′ 34.99″ E | 61m | Yesagyo | Magway | 7.68 |
| M024, M025 | 21° 14′ 57.08″ N  96° 1′ 25.59″ E | 116m | Wundwin | Mandalay | 7.42 | M085, M086 | 21° 36′ 45.70″ N  95° 12′ 32.29″ E | 63m | Yesagyo | Magway | 7.67 |
| M026, M027 | 21° 15′ 59.34″ N  96° 1′ 2.23″ E | 114m | Wundwin | Mandalay | 7.43 | M087 | 21° 36′ 44.24″ N  95° 12′ 35.63″ E | 63m | Yesagyo | Magway | 7.84 |
| M028, M029 | 21° 18′ 39.45″ N  96° 1′ 6.18″ E | 113m | Wundwin | Mandalay | 7.40 | M088, M089 | 21° 36′ 48.14″ N  95° 12′ 37.69″ E | 61m | Yesagyo | Magway | 7.81 |
| M030, M031 | 21° 19′ 31.45″ N  96° 1′ 40.44″ E | 108m | Wundwin | Mandalay | 7.41 | M090, M091 | 20° 13′ 38.15″ N  94° 48′ 0.32″ E | 45m | Minbu | Magway | 6.82 |
| M032, M033 | 21° 21′ 4.15″ N  96° 7′ 55.81″ E | 96m | Myittha | Mandalay | 7.82 | M092 | 20° 13′ 40.78″ N  94° 48′ 1.14″ E | 45m | Minbu | Magway | 6.84 |
| M034, M035 | 21° 21′ 6.37″ N  96° 8′ 32.41″ E | 96m | Myittha | Mandalay | 7.67 | M093, M094 | 20° 13′ 44.65″ N  94° 48′ 3.46″ E | 45m | Minbu | Magway | 7.30 |
| M036, M037 | 21° 22′ 39.07″ N  96° 10′ 25.83″ E | 97m | Kume | Mandalay | 7.88 | M095, M096 | 20° 13′ 43.03″ N  94° 48′ 11.33″ E | 45m | Minbu | Magway | 7.58 |
| M038, M039 | 21° 23′ 32.87″ N  96° 10′ 46.29″ E | 97m | Kume | Mandalay | 7.82 | M097, M098 | 20° 13′ 43.20″ N  94° 47′ 42.70″ E | 45m | Minbu | Magway | 7.24 |
| M040, M041 | 21° 47′ 27.92″ N  96° 4′ 15.89″ E | 73m | Kume | Mandalay | 7.71 | M099, M100 | 20° 14′ 23.32″ N  94° 45′ 34.86″ E | 48m | Minbu | Magway | 5.53 |
| M042 | 21° 56′ 16.28″ N  95° 54′ 20.05″ E | 66m | Sagaing | Sagaing | 7.52 | M101 | 20° 17′ 39.30″ N  94° 45′ 11.49″ E | 47m | Pwintphyu | Magway | 6.62 |
| M043 | 21° 56′ 24.58″ N  95° 53′ 31.35″ E | 72m | Sagaing | Sagaing | 7.40 | M102 | 20° 17′ 37.87″ N  94° 45′ 14.25″ E | 44m | Pwintphyu | Magway | 7.28 |
| M044 | 21° 56′ 20.66″ N  95° 53′ 27.61″ E | 72m | Sagaing | Sagaing | 7.36 | M103 | 20° 17′ 55.59″ N  94° 45′ 5.76″ E | 45m | Pwintphyu | Magway | 7.08 |
| M045, M046 | 21° 56′ 40.91″ N  95° 53′ 9.46″ E | 73m | Sagaing | Sagaing | 7.17 | M104, M105 | 20° 17′ 53.57″ N  94° 45′ 2.20″ E | 45m | Pwintphyu | Magway | 6.76 |
| M047 | 21° 56′ 38.26″ N  95° 53′ 9.39″ E | 74m | Sagaing | Sagaing | 7.35 | M106 | 20° 17′ 50.14″ N  94° 45′ 3.49″ E | 45m | Pwintphyu | Magway | 6.98 |
| M048 | 21° 56′ 34.06″ N  95° 53′ 5.07″ E | 73m | Sagaing | Sagaing | 7.48 | M107 | 20° 21′ 31.69″ N  94° 45′ 49.03″ E | 46m | Pwintphyu | Magway | 7.22 |
| M049, M050 | 21° 59′ 37.85″ N  95° 48′ 41.51″ E | 84m | Sagaing | Sagaing | 7.72 | M108 | 20° 21′ 14.53″ N  94° 45′ 53.86″ E | 45m | Pwintphyu | Magway | 7.53 |
| M051 | 21° 59′ 2.23″ N  95° 47′ 28.30″ E | 82m | Sagaing | Sagaing | 7.63 | M109, M110 | 20° 20′ 33.18″ N  94° 45′ 41.83″ E | 45m | Pwintphyu | Magway | 7.42 |
| M052 | 21° 59′ 7.90″ N  95° 47′ 28.47″ E | 83m | Sagaing | Sagaing | 7.68 | M111, M112 | 20° 18′ 42.68″ N  94° 45′ 26.88″ E | 42m | Pwintphyu | Magway | 7.38 |
| M053 | 21° 58′ 57.18″ N  95° 42′ 52.69″ E | 70m | Sagaing | Sagaing | 7.08 | M113, M114 | 20° 18′ 44.50″ N  94° 45′ 30.90″ E | 42m | Pwintphyu | Magway | 7.21 |
| M054 | 21° 59′ 28.19″ N  95° 42′ 5.56″ E | 65m | Sagaing | Sagaing | 7.60 | M115 | 20° 29′ 3.50″ N  94° 39′ 30.28″ E | 64m | Salin | Magway | 7.25 |
| M055, M056 | 21° 59′ 6.67″ N  95° 41′ 17.00″ E | 63m | Sagaing | Sagaing | 6.75 | M116 | 20° 31′ 7.71″ N  94° 39′ 35.74″ E | 67m | Salin | Magway | 7.55 |
| M057, M058 | 21° 58′ 58.63″ N  95° 39′ 15.42″ E | 69m | Myinmu | Sagaing | 6.62 | M117 | 20° 31′ 13.30″ N  94° 39′ 34.90″ E | 68m | Salin | Magway | 7.58 |
| M059, M060 | 21° 58′ 54.26″ N  95° 39′ 13.48″ E | 70m | Myinmu | Sagaing | 6.34 | M118, M119 | 20° 31′ 57.99″ N  94° 39′ 51.21″ E | 61m | Salin | Magway | 7.63 |
| M061 | 21° 59′ 9.77″ N  95° 38′ 48.60″ E | 71m | Myinmu | Sagaing | 7.05 | M120 | 20° 31′ 56.09″ N  94° 39′ 53.10″ E | 59m | Salin | Magway | 7.29 |
|  |  |  |  |  |  |  |  |  |  |  |  |

**Table S2.** Primers and PCR conditions for the genes studied

| **Gene** | **Primer sequence** | **PCR condition** | **Reference** |
| --- | --- | --- | --- |
| **16S-23S rDNA** | **FGPS-** (5´TGC GGC TGG ATC ACC TCC T3´)  **FGPL-132-** (5´CCG GGT TTC CCC ATT CGG3´) | 5 min at 95°C, 35 cycles of 30 s at 95°C, 30s at 56°C, 2 min at 72°C, and final extension at 72°C for 5 min | (Laguerre, et al. 1996: 2029-36) |
| ***nodC*** | ***nodC*MesoF-**(5´CGA(CT) CG(AG) AG(AG) TTC AA(CT) TTC3´)  ***nodC*MesoR-** (5´CT(CT) AAT GTA CAC A(AG) (GC)GC3´) | 1 min at 95°C, 35 cycles of 30 s at 95°C, 30 s at 46.5°C, 2 min at 68°C, and final extension at 68°C for 5 min | (Rivas, et al. 2007: 412-8) |
| ***nifH*** | ***nifH*-1-** (5'AAG TGC GTG GAG TCC GGT GG3')  ***nifH*-2**- (5'-GTT CGG CAA GCA TCT GCT CG-3') | 2 min at 95°C, and 35 cycles of 30 s at 95°C, 30 s at 61°C, 2 min at 68°C, and final extension at 68°C for 5 min | (Eardly, et al. 1992: 1809-15) |
| ***dnaJ*** | ***dnaJ*-F-** (5'CAG ATC GAG GTS ACC TTC GAC3')  ***dnaJ*-R-** (5'CGT CRY CAT MGA GAT CGG CAC3') | 5 min at 95°C, and 30 cycles of 45 s at 94°C, 90 s at 68°C, 2 min at 68°C | (Alexandre, et al. 2008: 2839-49) |
| ***recA*** | ***recA*-F1-** (5'CGK CTS GTA GAG GAY AAA TCG GTG GA3')  ***recA*-R1-** (5'CGR ATC TGG TTG ATG AAG ATC ACC AT3') | 5 min at 95°C, and 30 cycles of 45 s at 94°C, 60 s at 50°C, 90 s at 74°C | (Gaunt, et al. 2001: 2037-48) |

**Table S3**. GenBank accession numbers of rhizobial strains tested in this study

| **Strain** | **Accession number** | | | **Strain** | **Accession number** | | |
| --- | --- | --- | --- | --- | --- | --- | --- |
|  | **16S-23S rDNA IGS** | ***nodC*** | ***nifH*** |  | **16S-23S rDNA IGS** | ***nodC*** | ***nifH*** |
| M001 | MW712744 | MW736165 | MW736279 | M063 | MW712802 | MW736223 | MW736337 |
| M002 | MW712745 | MW736166 | MW736280 | M064 | MW712803 | MW736224 | MW736338 |
| M003 | MW712746 | MW736167 | MW736281 | M065 | MW712804 | MW736225 | MW736339 |
| M004 | MW712747 | MW736168 | MW736282 | M066 | MW712805 | MW736226 | MW736340 |
| M005 | MW712748 | MW736169 | MW736283 | M067 | MW712806 | MW736227 | MW736341 |
| M006 | MW712749 | MW736170 | MW736284 | M068 | MW712807 | MW736228 | MW736342 |
| M007 | MW712750 | MW736171 | MW736285 | M069 | MW712808 | MW736229 | MW736343 |
| M008 | MW712751 | MW736172 | MW736286 | M070 | MW712809 | MW736230 | MW736344 |
| M009 | MW712752 | MW736173 | MW736287 | M071 | MW712810 | MW736231 | MW736345 |
| M010 | MW712753 | MW736174 | MW736288 | M072 | MW712811 | MW736232 | MW736346 |
| M011 | MW712754 | MW736175 | MW736289 | M073 | MW712812 | MW736233 | MW736347 |
| M012 | MW712755 | MW736176 | MW736290 | M074 | MW712813 | MW736234 | MW736348 |
| M013 | MW712756 | MW736177 | MW736291 | M075 | MW712814 | MW736235 | MW736349 |
| M015 | MW712757 | MW736178 | MW736292 | M076 | MW712815 | MW736236 | MW736350 |
| M016 | MW712758 | MW736179 | MW736293 | M077 | MW712816 | MW736237 | MW736351 |
| M017 | MW712759 | MW736180 | MW736294 | M078 | MW712817 | MW736238 | MW736352 |
| M018 | MW712760 | MW736181 | MW736295 | M079 | MW712818 | MW736239 | MW736353 |
| M019 | MW712761 | MW736182 | MW736296 | M080 | MW712819 | MW736240 | MW736354 |
| M020 | MW712762 | MW736183 | MW736297 | M081 | MW712820 | MW736241 | MW736355 |
| M021 | MW712763 | MW736184 | MW736298 | M082 | MW712821 | MW736242 | MW736356 |
| M022 | MW712764 | MW736185 | MW736299 | M083 | MW712822 | MW736243 | MW736357 |
| M023 | MW712765 | MW736186 | MW736300 | M084 | MW712823 | MW736244 | MW736358 |
| M024 | MW712766 | MW736187 | MW736301 | M085 | MW712824 | MW736245 | MW736359 |
| M025 | MW712767 | MW736188 | MW736302 | M086 | MW712825 | MW736246 | MW736360 |
| M026 | MW712768 | MW736189 | MW736303 | M087 | MW712826 | MW736247 | MW736361 |
| M027 | MW712769 | MW736190 | MW736304 | M088 | MW712827 | MW736248 | MW736362 |
| M028 | MW712770 | MW736191 | MW736305 | M089 | MW712828 | MW736249 | MW736363 |
| M029 | MW712771 | MW736192 | MW736306 | M090 | MW712829 | MW736250 | MW736364 |
| M030 | MW712772 | MW736193 | MW736307 | M091 | MW712830 | MW736251 | MW736365 |
| M031 | MW712773 | MW736194 | MW736308 | M093 | MW712831 | MW736252 | MW736366 |
| M032 | MW712774 | MW736195 | MW736309 | M094 | MW712832 | MW736253 | MW736367 |
| M033 | MW712775 | MW736196 | MW736310 | M095 | MW712833 | MW736254 | MW736368 |
| M034 | MW712776 | MW736197 | MW736311 | M096 | MW712834 | MW736255 | MW736369 |
| M035 | MW712777 | MW736198 | MW736312 | M097 | MW712835 | MW736256 | MW736370 |
| M036 | MW712778 | MW736199 | MW736313 | M098 | MW712836 | MW736257 | MW736371 |
| M037 | MW712779 | MW736200 | MW736314 | M099 | MW712837 | MW736258 | MW736372 |
| M038 | MW712780 | MW736201 | MW736315 | M100 | MW712838 | MW736259 | MW736373 |
| M039 | MW712781 | MW736202 | MW736316 | M101 | MW712839 | MW736260 | MW736374 |
| M040 | MW712782 | MW736203 | MW736317 | M102 | MW712840 | MW736261 | MW736375 |
| M041 | MW712783 | MW736204 | MW736318 | M103 | MW712841 | MW736262 | MW736376 |
| M042 | MW712784 | MW736205 | MW736319 | M104 | MW712842 | MW736263 | MW736377 |
| M043 | MW712785 | MW736206 | MW736320 | M105 | MW712843 | MW736264 | MW736378 |
| M044 | MW712786 | MW736207 | MW736321 | M106 | MW712844 | MW736265 | MW736379 |
| M045 | MW712787 | MW736208 | MW736322 | M107 | MW712845 | MW736266 | MW736380 |
| M046 | MW712788 | MW736209 | MW736323 | M108 | MW712846 | MW736267 | MW736381 |
| M047 | MW712789 | MW736210 | MW736324 | M109 | MW712847 | MW736268 | MW736382 |
| M048 | MW712790 | MW736211 | MW736325 | M110 | MW712848 | MW736269 | MW736383 |
| M050 | MW712791 | MW736212 | MW736326 | M111 | MW712849 | MW736270 | MW736384 |
| M051 | MW712792 | MW736213 | MW736327 | M112 | MW712850 | MW736271 | MW736385 |
| M053 | MW712793 | MW736214 | MW736328 | M113 | MW712851 | MW736272 | MW736386 |
| M054 | MW712794 | MW736215 | MW736329 | M114 | MW712852 | MW736273 | MW736387 |
| M055 | MW712795 | MW736216 | MW736330 | M116 | MW712853 | MW736274 | MW736388 |
| M056 | MW712796 | MW736217 | MW736331 | M117 | MW712854 | MW736275 | MW736389 |
| M057 | MW712797 | MW736218 | MW736332 | M118 | MW712855 | MW736276 | MW736390 |
| M058 | MW712798 | MW736219 | MW736333 | M119 | MW712856 | MW736277 | MW736391 |
| M059 | MW712799 | MW736220 | MW736334 | M120 | MW712857 | MW736278 | MW736392 |
| M061 | MW712800 | MW736221 | MW736335 | CC1192 | MW584799 | MW676866 | MW676943 |
| M062 | MW712801 | MW736222 | MW736336 |  |  |  |  |

**Table S4.** Differential phenotypic characteristics of 16 selected strains from Australia and Myanmar, and closely related species of *Mesorhizobium.*

| **Strains** | **SE** | **AT** | **HT** | **ST** | **Host** | **Origin** | **References** |
| --- | --- | --- | --- | --- | --- | --- | --- |
| A10 | - | + | + | - | chickpea | Australia | This study |
| A14 | - | + | - | - | chickpea | Australia | This study |
| A21 | + | + | - | - | chickpea | Australia | This study |
| A47 | + | + | - | - | chickpea | Australia | This study |
| A53 | + | + | + | - | chickpea | Australia | This study |
| A54 | + | + | - | + | chickpea | Australia | This study |
| A68 | + | + | - | + | chickpea | Australia | This study |
| A78 | + | + | - | - | chickpea | Australia | This study |
| M009 | + | - | - | - | chickpea | Myanmar | This study |
| M021 | - | - | + | + | chickpea | Myanmar | This study |
| M062 | - | + | + | + | chickpea | Myanmar | This study |
| M075 | + | + | **+** | + | chickpea | Myanmar | This study |
| M082 | + | - | + | + | chickpea | Myanmar | This study |
| M094 | + | + | + | + | chickpea | Myanmar | This study |
| M107 | + | + | + | + | chickpea | Myanmar | This study |
| M113 | - | + | + | + | chickpea | Myanmar | This study |
| M. *ciceri* CC1192 | + | + | - | + | chickpea | Israel | This study |
| *M. temperatum* |  | + | + | + | *Astragalus adsurgens* | China | (Gao, et al. 2004: 2003-12) |
| *M. septentrionale* |  | + | - | + | *Astragalus adsurgens* | China | (Gao, et al. 2004: 2003-12) |
| *M. tianshanense* |  | **-** | - | + | wild legume | China | (Chen, et al. 1995: 153-9, Tan, et al. 1997: 874-9) |
| *M. amorphae* |  | + | - | + | *Amorpha fruticosa* | China | (Wang, et al. 1999: 51-65) |
| *M. gobiense* |  | - | + | + | wild legume | China | (Han, et al. 2008: 2610-8) |
| *M. tarimense* |  | **-** | + | + | wild legume | China | (Han, et al. 2008: 2610-8) |
| *M. silamurunense* |  | **-** | + | - | *Astragalus* spp. | China | (Zhao, et al. 2012: 2180-6) |
| *M. ciceri* |  | + | - | - | chickpea | Spain | (Nour, et al. 1994: 345-54) |
| *M. mediterraneum* |  | - | + | - | chickpea | Spain | (Nour, et al. 1995: 640-8) |
| *M. loti* |  | + | - | **-** | *Lotus corniculatus* | New Zealand | (Jarvis, et al. 1982: 378-80) |
| *M. opportunistum* |  | + | + | **+** | *Biserrula pelecinus* | Australia | (Nandasena, et al. 2009: 2140-7) |
| *M. plurifarium* |  | + | + | + | *Acacia senegal* | Senegal | (de Lajudie, et al. 1998: 369-82) |
| *M. tamadayense* |  | **+** | + | + | *Anagyris latifolia* | Spain | (Ramírez-Bahena, et al. 2012: 334-41) |
| *M. metallidurans* |  | + | + | - | *Anthyllis vulneraria* | France | (Vidal, et al. 2009: 850-5) |
| *M. huakuii* |  | + | + | **+** | *Astragalus sinicus* | China | (Chen, et al. 1991: 275-80) |
| *M. caragnae* |  | + | - | - | *Caragana spp.* | China | (Guan, et al. 2008: 2646-53) |

Note: SE, symbiotic effectiveness (>80%); AT, acid tolerance (≥5); HT, heat tolerance (37, 40 ºC); ST, salt tolerance (≤2% NaCl)


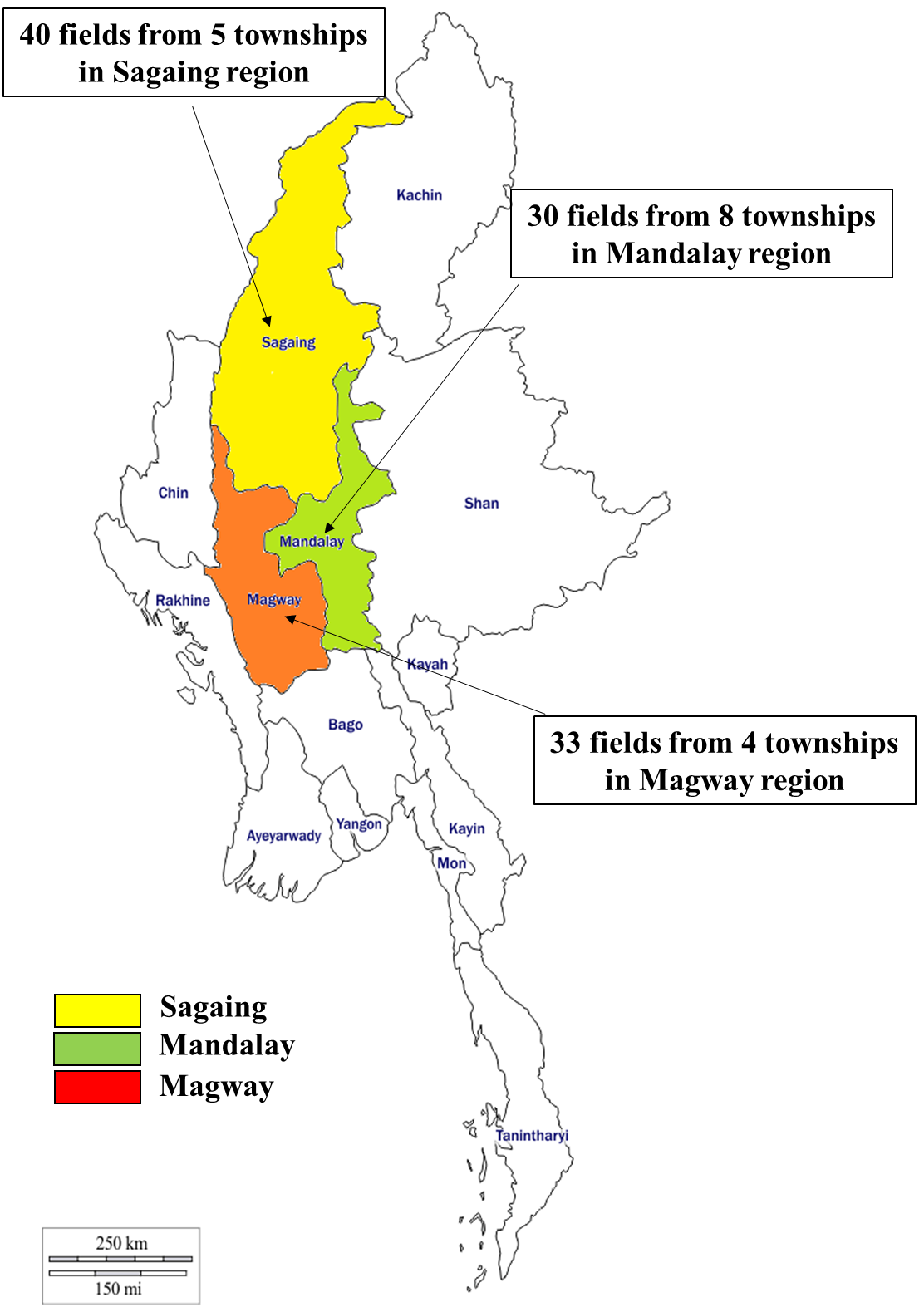


**Figure S1**. Map showing the sample collection sites in the Central Dry Zone of Myanmar.


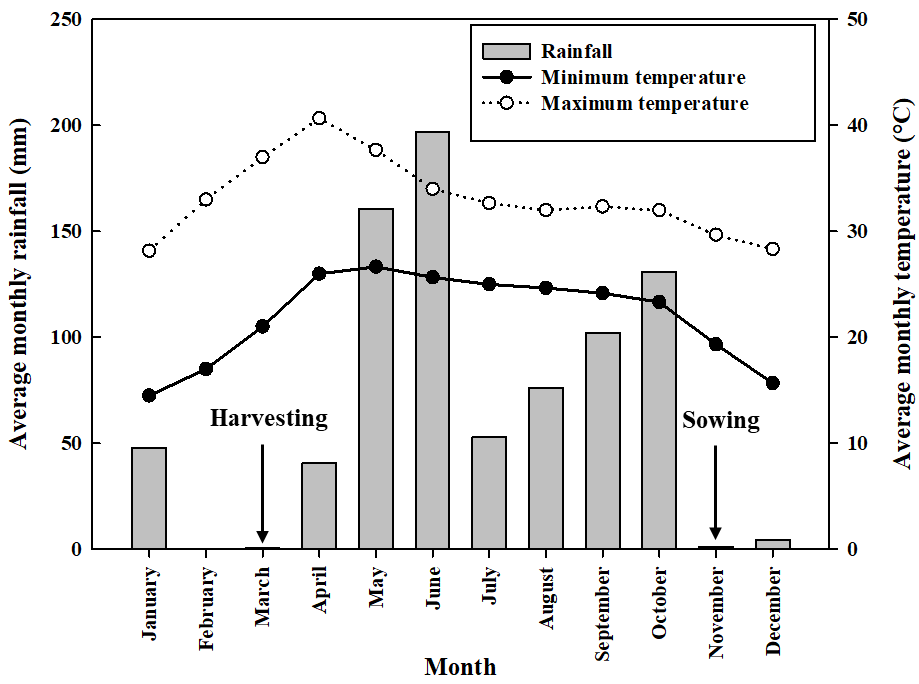


**Figure S2.** Monthly rainfall distribution and temperatures across soil sampling sites during the 2018 growing season in the Central Dry Zone, Myanmar. (Source: Department of Meteorology and Hydrology,Naypyidaw, Myanmar).


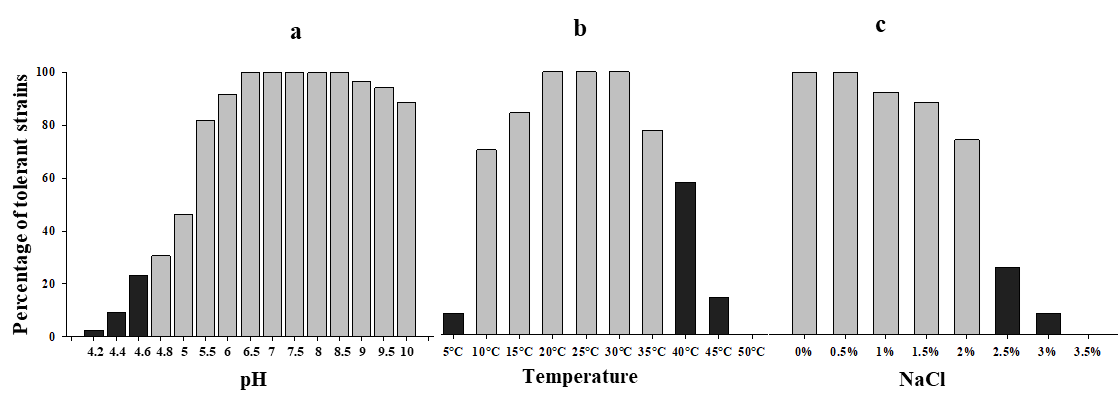


**Figure S3.** Percentage of tolerant strains (120 test strains and strain CC1192) to (a) pH, (b) temperature (°C) and (c) NaCl (% w/v). Grey bars show the percentage of tolerant strains including strain CC1192; black bars indicate the percentage of strains that were tolerant but where strain CC1192 was unable to grow.


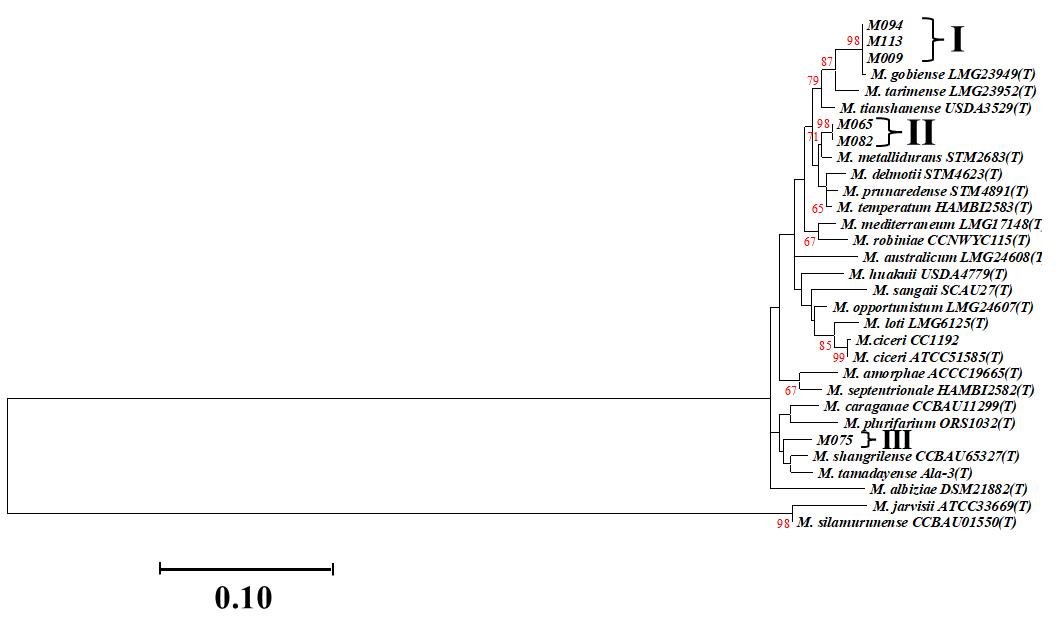


**Figure S4.** Phylogenetic trees based on partial sequences of *dnaJ* (a) and *recA* (b) showing the relatedness of chickpea rhizobial strains and recognized species of *Mesorhizobium*. The Maximum Likelihood phylogenetic tree was constructed using Kimura 2-parameter model MEGA7. The percentage of trees in which the associated taxa clustered together was presented next to the branches. Bootstrap values were computed based on 1000 replications. The scale bar (0.1) represents the percentage of nucleotide substitution per site. All positions containing gaps and missing data were eliminated. The groups were generated according to 16S-23S rDNA-based phylogeny. *M*, *Mesorhizobium*.
